# DeepMDSCBA: An Improved Semantic Segmentation Model Based on DeepLabV3+ for Apple Images

**DOI:** 10.3390/foods11243999

**Published:** 2022-12-10

**Authors:** Lufeng Mo, Yishan Fan, Guoying Wang, Xiaomei Yi, Xiaoping Wu, Peng Wu

**Affiliations:** 1 College of Mathematics and Computer Science, Zhejiang A&F University, Hangzhou 311300, China; 2 School of Information Engineering, Huzhou University, Huzhou 313000, China

**Keywords:** apple image, semantic segmentation, convolutional block attention module, depthwise separable convolution

## Abstract

The semantic segmentation of apples from images plays an important role in the automation of the apple industry. However, existing semantic segmentation methods such as FCN and UNet have the disadvantages of a low speed and accuracy for the segmentation of apple images with complex backgrounds or rotten parts. In view of these problems, a network segmentation model based on deep learning, DeepMDSCBA, is proposed in this paper. The model is based on the DeepLabV3+ structure, and a lightweight MobileNet module is used in the encoder for the extraction of features, which can reduce the amount of parameter calculations and the memory requirements. Instead of ordinary convolution, depthwise separable convolution is used in DeepMDSCBA to reduce the number of parameters to improve the calculation speed. In the feature extraction module and the cavity space pyramid pooling module of DeepMDSCBA, a Convolutional Block Attention module is added to filter background information in order to reduce the loss of the edge detail information of apples in images, improve the accuracy of feature extraction, and effectively reduce the loss of feature details and deep information. This paper also explored the effects of rot degree, rot position, apple variety, and background complexity on the semantic segmentation performance of apple images, and then it verified the robustness of the method. The experimental results showed that the PA of this model could reach 95.3% and the MIoU could reach 87.1%, which were improved by 3.4% and 3.1% compared with DeepLabV3+, respectively, and superior to those of other semantic segmentation networks such as UNet and PSPNet. In addition, the DeepMDSCBA model proposed in this paper was shown to have a better performance than the other considered methods under different factors such as the degree or position of rotten parts, apple varieties, and complex backgrounds.

## 1. Introduction

Computer vision is widely used in digital agriculture [1]. As a basic task in computer vision, semantic segmentation is applied in all aspects of agricultural automation. Semantic segmentation technology also provides strong technical support for the work of precision agricultural robots. The semantic segmentation of crop fruits can help robots detect and locate fruit positions and realize automatic picking, thereby reducing manual participation in agriculture, improving agricultural efficiency, and reducing production costs.

A widely used semantic segmentation network is the Fully Convolutional Network (FCN) proposed by Long et al. [2]. Wang et al. used the FCN to realize the recognition of wheat ear targets that is difficult to achieve with traditional methods [3]. The Pyramid Scene Parsing Network (PSPNet) [4] has made some improvements based on the FCN, adding an encoder–decoder structure that allows for more refined deconvolution results, improved classification accuracy, and improved overall efficiency [5]. Deng et al. [6] used PSPNet to segment a range of kiwifruit vines based on kiwifruit orchard images. The DeepLab semantic segmentation method uses Atrous Spatial Pyramid Pooling (ASPP), which can expand the receptive field without changing the resolution; at the same time, the features of different levels can be fused [7]. DeepLabV3+ as found to have better segmentation results than former segmentation methods such as FCN and PSPNet. Zhang et al. used DeepLabV3+ to segment the lodging area of different wheat growth stages, and their segmentation accuracy was high [8]. Using DeepLabV3+, Sharifzadeh et al. detected farm crops with low-resolution satellite images, and their edge segmentation effect was better [9].

However, the DeepLabV3+ method also has some shortcomings. Firstly, its computational complexity is relatively high because its feature extraction network Xception has a large number of network layers and a large number of parameters and the convolution method in the ASPP module is ordinary convolution, which increases the number of parameters. Secondly, its feature information extraction can be improved. In the process of feature extraction at the encoder, the spatial dimension of the input data is gradually reduced, resulting in the loss of useful information, and detail recovery cannot be achieved well during decoding. Finally, the target edge recognition accuracy is relatively low. Although the ASPP module can improve the method’s ability to extract the boundary of a target, it cannot fully simulate the relationship between the local features of a target, resulting in a reduction in the accuracy of the target segmentation and subsequent problems such as a low recognition accuracy and poor edge recognition.

In view of the above issues, in order to obtain more efficiently and accurately achieve apple fruit segmentation, DeepMDSCBA (a network segmentation model based on DeepLabV3+ with the MobileNet, Depthwise Separable Convolution, and Convolutional Block Attention modules) is proposed in this paper. DeepMDSCBA was constructed based on the DeepLabV3+ structure, and a more lightweight MobileNet network [10] was adopted as its backbone network other than the original Xception network to reduce the amount of parameter calculations and memory usage. In DeepMDSCBA, Depthwise Separable Convolution (DSC) [11] is used to replace the ordinary convolution in the ASPP module to improve the calculation speed of the method. In the feature extraction and ASPP modules of DeepMDSCBA, a Convolutional Block Attention Module (CBAM) [12] is added to filter the background information and reduce the loss of image edge detail information. Because of the improvements in the above aspects, the information processing efficiency and accuracy of DeepMDSCBA were found to be improved and the accuracy of the segmentation model was increased.

In addition, to verify the robustness of DeepMDSCBA, the influence of rot degree, rot position, apple variety, and complexity of background on the performance of apple image semantic segmentation were extensively studied in this paper.

The rest of this article is organized as follows. Section 2 describes the main idea of the DeepMDSCBA method. Section 3 describes the design and setup of our experiments. Section 4 analyzes the results of the comparative experiments. Finally, the last section summarizes the work.

## 2. DeepMDSCBA Segmentation Method

### 2.1. Main Ideas

**Figure 1:**
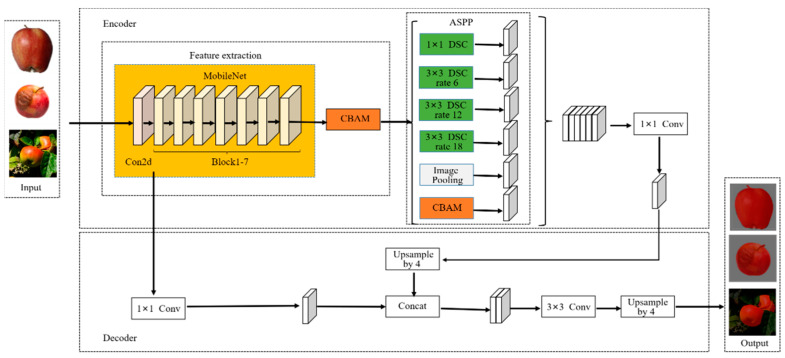
Structure of DeepMDSCBA method.

The proposed DeepMDSCBA method, a network segmentation method based on deep learning, is demonstrated in Figure 1.

According to Figure 1, the main ideas of DeepMDSCBA are as follows:

(1) Using MobileNet to replace Xception: Based on the DeepLabV3+ framework, the backbone Xception network for method feature extraction was replaced with a more lightweight MobileNet to greatly reduce the amount of parameter calculations, reduce memory usage, and improve the calculation speed of the method.
(2) Using DSC to replace ordinary convolution: The ordinary convolution in the ASPP module was changed to DSC to further reduce the method’s parameters and improve the method’s calculation speed.
(3) Adding CBAM: CBAM was added to the feature extraction module and the ASPP module to reduce accuracy loss in order to train a more accurate segmentation method and improve the segmentation accuracy of the proposed model for apple images.

### 2.2. Using MobileNet to Replace Xception

DeepLabV3+ is an improved version of DeepLabV3. It replaces the underlying network with a residual network and adds an encoding–decoding structure to restore spatial information to obtain clear object boundaries so that boundary segmentation is optimized [13,14]. At the encoder end, DeepLabV3+ uses the Xception network to extract the features of the input image, and it then uses the ASPP module to fuse image features to avoid information loss. Xception is a deep convolutional neural network with an input stream, an intermediate stream, and an output stream, and ASPP is a multi-scale pyramid feature extraction module. At the decoder end, the low-level features from the Xception module and the high-level features from the encoder are fused, and then bilinear interpolation upsampling is executed to output the segmentation results, which finally improves the accuracy of network segmentation.

In DeepMDSCBA, the method proposed in this paper, MobileNet (a more lightweight network) was adopted to replace Xception, the original backbone of DeepLabV3+. Compared with the Xception network, MobileNet has a shallower network layer, fewer parameters, a lower method complexity, a faster network training speed, and a faster convergence. This replacement solves the problems of large convolutional neural networks and insufficient hardware training in the process of image method training. In the version of the implementation of DeepMDSCBA in this paper, the second version of MobileNet, MobileNetV2, is used. This version mainly consists of three parts. The first part is a 3 × 3 convolution block for feature extraction, the second part is a network structure containing multiple DSC layers composed of multiple 1 × 1 and 3 × 3 convolution blocks, and the last part consists of two 1 × 1 convolution blocks and a 7 × 7 average pooling block [15]. Combined with the DeepLabV3+ network structure, DeepMDSCBA uses the front two parts of the network, and the specific network structure is shown in Table 1.

### 2.3. Using DSC to Replace Ordinary Convolution

The main function of a convolution layer is feature extraction. In the process of the convolution of the input feature map through the convolution kernel, the spatial features and channel features need to be learned at the same time while the deep separable convolution decouples the spatial correlation and channel correlation of the convolution layer, adds a transition layer in the standard convolution process, and decomposes it into depthwise convolution [16] and pointwise convolution [17] considering spatial correlation and channel correlation, respectively. Compared with standard convolution, DSC can greatly reduce the number of parameters and calculations while ensuring little loss of accuracy. 

**Figure 2:**
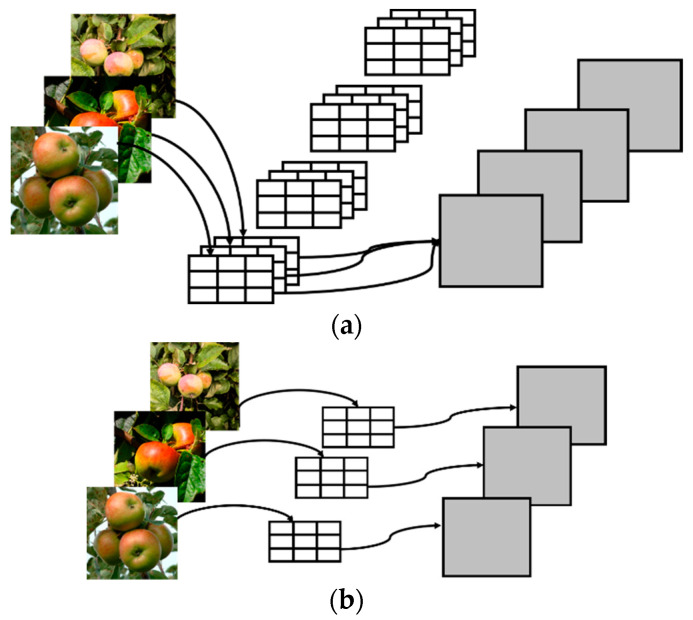

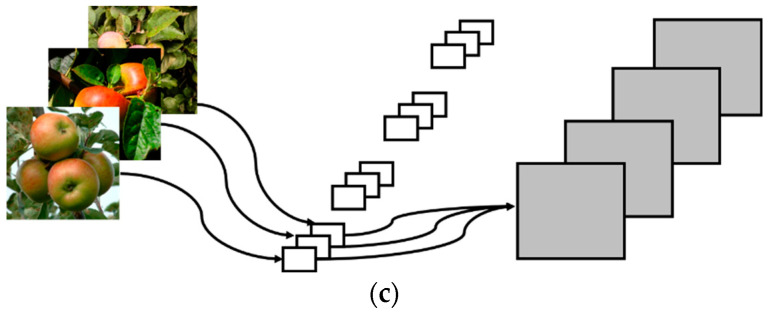
Standard convolution and depthwise separable convolution operation. (**a**) Standard convolution process. (**b**) Depthwise convolution process. (**c**) Pointwise convolution process.

Suppose the input is a feature map of $D_{F}\times D_{F}\times M$ and it is convoluted with a convolution kernel of $D_{K}\times D_{K}\times M\times N$ in size; the standard convolution operation process is shown in Figure 2a. Each input feature map is convoluted with M× $D_{K}\times D_{K}$ convolution kernels in the N class and then summed and biased to obtain an output. The final output is $D_{F}\times D_{F}\times N$ in size. The depthwise separable operation process is shown in the following figure. Figure 2b shows the depthwise convolution process, and Figure 2c shows the pointwise convolution process.

In depthwise convolution, each input feature map is only biased by convolution with the corresponding convolution kernel, and the output size is $D_{F}\times D_{F}\times M$. In point-by-point convolution, the feature map of $D_{F}\times D_{F}\times M$ is standardly convoluted with N × 1 × 1 convolution kernels, and the number of channels is changed. Finally, the output feature map size is $D_{F}\times D_{F}\times N$.

The standard convolution process calculation is as follows.

(1) $$D_{F}\times D_{F}\times A=\pi r^{2}\times N\times D_{K}\times D_{K}$$

In depthwise separable convolution, the calculation amount of the depthwise convolution process is as follows.

(2) $$D_{F}\times D_{F}\times M\times D_{K}\times D_{K}$$

The calculation amount of pointwise convolution process is as follows.

(3) $$D_{F}\times D_{F}\times M\times N$$

The depthwise separable convolution is equivalent to n times the standard convolution. The expression of n is as follows.

(4) $$\frac{D_{F}\times D_{F}\times M\times D_{K}\times D_{K}+D_{F}\times D_{F}\times M\times N}{D_{F}\times D_{F}\times M\times N\times D_{K}\times D_{K}}=\frac{1}{N}+\frac{1}{D_{K}\times D_{K}}$$

In above formulas, $D_{F}\times D_{F}$ is the size pixel of input feature map, M is the number of input channels, $D_{K}\times D_{K}$ is the size pixel of convolution kernel, and N is the number of output channels.

### 2.4. Adding CBAM

**Figure 3:**
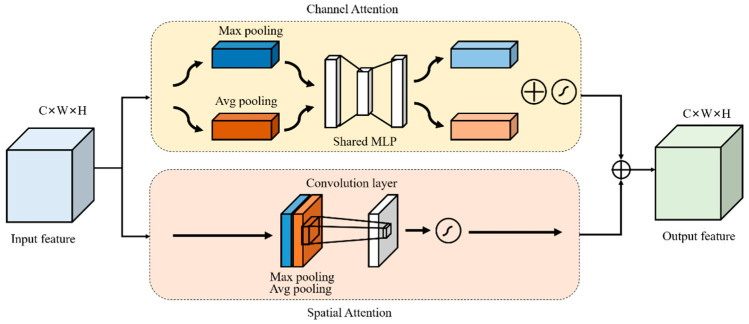
Structure of CBAM.

Attention plays an important role in human perception. Humans use a series of local observations and selectively focus on salient parts to better capture visual structures. In recent years, many researchers have improved the performance of convolutional neural networks in large-scale classification tasks by adding attention mechanisms. In this paper, CBAM was added to the feature extraction and ASPP modules. Firstly, the global dependencies between features are captured in the spatial and channel dimensions of features to capture the context feature information and enhance the expression ability of features, respectively. Then, the outputs of the two attention modules are added to further improve the feature representation; finally, more accurate segmentation results can be obtained [18]. The structure of CBAM is shown in Figure 3.

CBAM is a lightweight convolutional attention module that combines the channel attention mechanism and the spatial attention mechanism.

The channel attention mechanism selectively emphasizes interconnected channel maps by integrating relevant features in all channel maps [19]. Therefore, DeepMDSCBA explicitly models the interdependence between channels by adding a channel attention mechanism module. Assume an input feature map $F\in R^{C\times W\times H}$ (where C denotes the number of channels of the input feature map and W and H denote the width and height of the feature map, respectively). Firstly, the parallel global maximum pooling and global average pooling are used to compress the spatial dimension of the input feature map *F* to obtain the background description $F_{max}^{c}$ and $F_{avg}^{c}$, respectively. Then, the sigmoid function is added by the shared network calculation composed of multi-layer perceptron MLP to obtain the channel attention mechanism mapping feature map $M_{C}\in R^{C\times 1\times 1}$. Finally, the generated feature map is mapped to C×W×H, and the original input feature map is multiplied by elements to obtain the channel attention-weighted graph $F_{Cout}\in R^{C\times W\times H}$. The specific calculation process is as follows:

(5) $$M_{C}=\sigma \left(MLP\left(F_{\max}^{c}\right)+MLP\left(F_{avg}^{c}\right)\right)$$

(6) $$F_{Cout}=F⊗F_{c}\left(M_{c}\right)$$

where σ is the sigmoid activation function, ⊗ is the multiplication between elements, and $F_{c}$ is the feature vector of C×W×H obtained by copying $M_{c}$ along the spatial dimension. 

The spatial attention mechanism selectively aggregates the features of each location through the weighted sum of the features of all locations. Similar features are interrelated regardless of distance [20,21,22]. Therefore, in order to establish a richer context relationship between local features, the spatial attention mechanism module is introduced. Assume an input feature map $F\in R^{C\times W\times H}$, where C denotes the number of channels of the input feature map and W and H denote the width and height of the feature map, respectively. Firstly, the background descriptions $F_{max}^{c}$ and $F_{avg}^{c}$ are obtained via the channel compression of the input feature map F using parallel global maximum pooling and global average pooling, respectively. The two feature maps are merged and the dimension is reduced to 1 channel by a 7 × 7 convolution operation. Then, the mapping feature map $M_{s}\in R^{1\times W\times H}$ of the spatial attention mechanism is obtained with the sigmoid function. Finally, the generated feature map is mapped to C×W×H, and the original input feature map is multiplied by elements to obtain a spatial attention-weighted graph $F_{Sout}\in R^{C\times W\times H}$. The specific calculation process is as follows:

(7) $$M_{s}=\sigma \left(f^{7\times 7}\left(\left[F_{max}^{s};F_{avg}^{s}\right]\right)\right)$$

(8) $$F_{Sout}=F⊗F_{s}\left(M_{s}\right)$$

where σ represents the sigmoid activation function, $f^{7\times 7}$ represents the convolution operation, the convolution kernel size is 7×7, ⊗ represents the multiplication between elements, and $F_{s}$ represents the C×W×H feature vector obtained by copying $M_{s}$ along the channel direction. 

## 3. Experiments

This section introduces the design of experiments to test the performance of DeepMDSCBA, the method proposed in this paper. First, the hardware and software for the equipment configuration of the experiments is introduced, then the dataset production and preprocessing required for the experiments are described, and the network hyperparameters are established. The designs of the robust and ablation experiments are introduced last. 

### 3.1. Hardware and Software Configuration

The experiments in this paper used the PyTorch deep learning framework to train and test the performance of the DeepMDSCBA method. The specific configuration of the experiments is shown in Table 2. 

### 3.2. Data Acquisition and Preprocessing

The images in the datasets used for the experiments were mainly obtained from the internet, together with image synthesis, which was divided into training and test sets. The training and test sets had no duplicate images. The training and test sets were classified according to different apple varieties, different degrees of rot, different positions of rot, and background complexity. After enhancing the training set through the addition of random rotation, noise and mirroring and increasing the sample size to improve the model’s generalization ability, we trained the model. Furthermore, the apple images in the datasets were labeled with the graphical interface labeling software LabelMe [23] to generate JSON files. Although DeepLabV3+ does not limit image parameters such as the resolution of the images in the dataset, images in the datasets were uniformly converted into grayscale images with a resolution of 512 × 512 and a depth of 24 and then were stored in the PASCAL VOC [24] data format, which allowed the comparison of the performance of DeepMDSCBA and that of various other methods.

### 3.3. Dataset Partitioning

#### 3.3.1. Dataset of Apple Images with Different Rot Degrees

**Figure 4:**
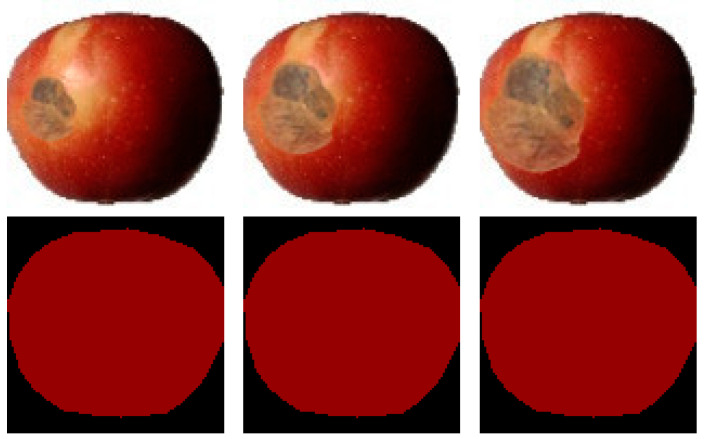
Apple images with different degrees of rot and their labels.

This dataset consists of apple images with different rot degrees, where the degree is expressed as the proportion of rotten area. All images in the dataset are apples in the front view. Due to the small number of naturally rotten apple images and the difficulty in controlling the degree and position of rot, in addition to the naturally rotten apple images, image synthesis technology was used to synthesize the rotten parts of apple images so that apple images with different rotten areas could be obtained for experiments. According to the ratio of the rotten area in the image to the entire apple area, the proportions of the rotten area to the apple image in the dataset were divided into five sub-datasets of (0, 20%], (20%, 40%], (40%, 60%], (60%, 80%], and (80%, 100%]. Some samples are shown in Figure 4.

#### 3.3.2. Dataset of Apple Images with Different Rot Positions

**Figure 5:**
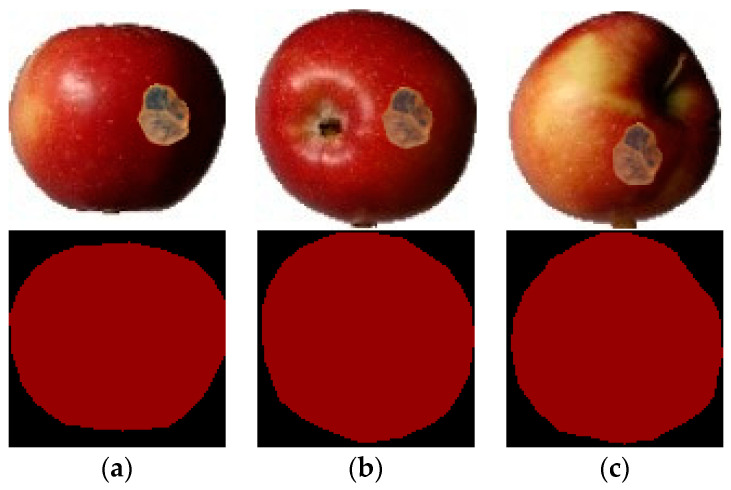
Apple images with different rot positions and their labels. (**a**) View without the stalk or calyx. (**b**) View of calyx. (**c**) View of stalk.

This dataset consists of three different views of apples. The views include the fruit stalk, fruit calyx, and neither fruit stalk nor fruit calyx. In order to unify the standard, an apple image with the proportion of rotten area between (0, 40%] was selected, and the dataset was divided into sub-datasets of the three abovementioned views to explore the influence of different rot positions on the apple semantic segmentation results. Some samples are shown in Figure 5.

#### 3.3.3. Dataset of Apple Images of Different Varieties

**Figure 6:**
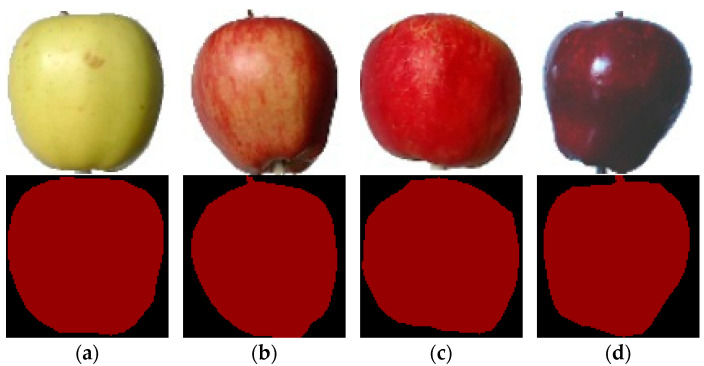
Images and labels of different apple varieties. (**a**) Golden Delicious. (**b**) Fuji. (**c**) Honey Crisp. (**d**) Red Delicious.

This dataset consists of images of different varieties of apples. Images of four common apple varieties—Golden Delicious, Fuji, Honey Crisp and Red Delicious—were selected for the experiments to explore the influence of different apple varieties on the segmentation results. Some samples are shown in Figure 6.

#### 3.3.4. Dataset of Apple Images with Complex Backgrounds

**Figure 7:**
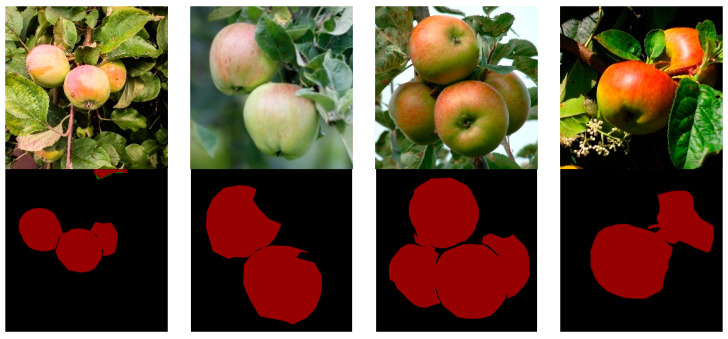
Apple images with complex backgrounds and their labels.

This dataset is mainly composed of multiple apple images in the natural state. The images are apples under relatively complex backgrounds with branches and leaves, which were used to explore the effect of complex backgrounds on the semantic segmentation of apple images using DeepMDSCBA in this study. Some samples of the dataset are shown in Figure 7.

### 3.4. Evaluation Indicators

In the experiments, MIoU (Mean Intersection over Union) and PA (Pixel Accuracy) were used as the evaluation indicators for apple image segmentation to analyze the segmentation performance.

(1) Pixel Accuracy (PA)

PA is the ratio of correctly predicted pixels to total pixels. The calculation formula is as follows:

(9) $$\mathrm{PA}=\frac{\sum_{i=0}^{k}P_{ii}}{\sum_{i=0}^{k}\sum_{j=0}^{k}P_{ij}}$$

In the formula, k denotes the total number of categories, $P_{ij}$ denotes the number of pixels that belong to class i but are predicted to belong to class $j,\;P_{ii}$ denotes the number of correctly predicted pixels, and $P_{ij}$ and $P_{ji}$ denote false positive and false negative results, respectively.

(2) Mean Intersection over Union (MIoU)

MIoU is the most commonly used metric in semantic segmentation experiments. It is used to calculate the ratio of the intersection and union of two sets of real and predicted values on each class and then to calculate the average value of the intersection and union ratio of all classes, that is, the average intersection and union ratio. The calculation formula is as follows:

(10) $$\mathrm{MIoU}=\frac{1}{k+1}\sum_{i=0}^{k}\frac{P_{ii}}{\sum_{j=0}^{k}P_{ij}+\sum_{j=0}^{k}P_{ji}-P_{ii}}$$

In the formula, k denotes the total number of categories, $P_{ij}$ denotes the number of pixels that belong to class i but are predicted to belong to class $j,\;P_{ii}$ denotes the number of correctly predicted pixels, and $P_{ij}$ and $P_{ji}$ denote false positive and false negative results, respectively.

### 3.5. Experimental Scheme

#### 3.5.1. Determination of Training Parameters

For the original DeepLabV3+ method, with an initial learning rate of 0.007 and a batch size of 16, the average intersection–union ratios of the method on the PASCAL VOC2012 and Cityscapes [25] datasets were 89.1% and 83.2%, respectively, achieving good segmentation results. On this basis, according to the commonly used empirical values of network training hyperparameters, and after repeated testing, the network hyperparameters of DeepMDSCBA used in the experiments were established. They are shown in Table 3.

#### 3.5.2. Test Scheme

In order to test the performance of the proposed DeepMDSCBA method in an apple image segmentation task, it was compared with the traditional semantic segmentation methods of FCN, SegNet, PSPNet, UNet [26] and DeepLabV3+. MIoU and PA were selected as indicators to test the segmentation performance of each method.

To test the segmentation efficiency of each method, the training time and single image prediction time, memory occupancy, and parameter quantity were selected as indicators.

In order to test the generalization ability of DeepMDSCBA and verify its robustness, segmentation and comparison experiments were performed on the constructed training and test sets, which comprised datasets of apple images with different levels of rot, different rot positions, different apple varieties, and complex backgrounds.

In order to verify the effectiveness of the ideas of DeepMDSCBA, such as adopting a more lightweight network (MobileNet) than the original feature extraction network (Xception), changing the ordinary convolution to DSC in the ASPP module, and adding CBAM to the feature extraction module and the ASPP module, the following ablation experiments were performed on the total test set.

(1) DeepM: Based on the traditional DeepLabV3+ network, the feature extraction network was changed to a more lightweight MobileNetV2 network.
(2) DeepMDS: On the basis of DeepM, the ordinary convolution in the ASPP module was changed to DSC.
(3) DeepMCBA: On the basis of DeepM, CBAM was added to the feature extraction and ASPP modules.
(4) DeepMDSCBA1: Based on DeepMDS, only CBAM was added to the feature extraction module.
(5) DeepMDSCBA2: Based on DeepMDS, only CBAM was added to the ASPP module.
(6) DeepMDSCBA: Based on DeepMDS, CBAM was added to the feature extraction and ASPP modules, which reflected the method proposed in this paper.

#### 3.5.3. Dataset Configuration

The training set and the test set adopted completely different pictures with no intersection.

The training set comprised a dataset of 212 images of fully healthy apples of different varieties without any rot, a dataset of 240 images of apples with different degrees of rot, a dataset of 180 images of apples with different positions of rot, and a dataset of 216 images of apples with complex backgrounds. The training set details are shown in Table 4.

For the three experiments related to the unseen cases described in 4.3.5, the training set for each experiment does not contain the corresponding case in the test set as an unseen case, that is a rot degree of (40%, 60%], a rot position of the calyx view, and a variety of Honey Crisp, respectively, as can be seen in Table 5. 

The test set was divided into four subsets: a dataset of 120 images of fully healthy apples of different varieties without any rot, a dataset of 200 images of apples with different degrees of rot, a dataset 90 images of apples with different rot positions, and a dataset of 50 images of apples with complex backgrounds. The sum of all test sets was the total test set. There were no repeated pictures in the test set, and apple image appeared multiple times. The details of the test set are shown in Table 5.

## 4. Results and Analysis

### 4.1. Performance of Segmentation

**Figure 8:**
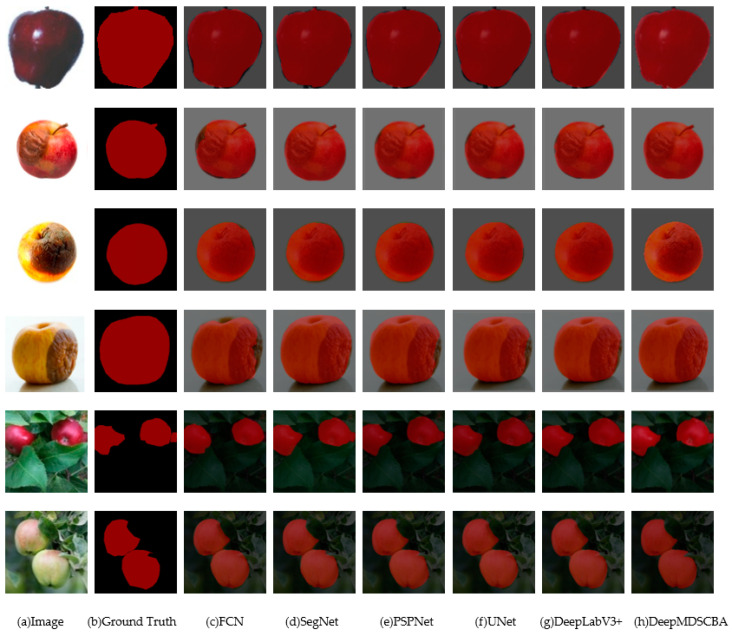
Results of segmentation using different algorithms.

In order to verify the segmentation performance of the DeepMDSCBA model, the model trained by the training set was used to perform segmentation tests on the previously divided total test set. In the experiment, the FCN, SegNet, PSPNet, UNet and DeepLabV3+ methods were used for comparison with the proposed DeepMDSCBA method. Some segmentation results are shown in Figure 8.

It can be seen in Figure 8 that compared with the other five methods, DeepMDSCBA showed the highest degree of recognition of the edges of apples with complex backgrounds in the images, as well as fewer omissions and misclassifications, especially for the rotten parts of apples.

Using MIoU and PA as indicators, the segmentation performance of apple images with DeepMDSCBA and the other five methods were analyzed, and the results are shown in Table 6.

It can be seen in Table 6 that the MIoU of DeepMDSCBA was 87.1%, which was 5.5%, 3.1%, 3.5%, 4.1% and 3.4% higher than that of FCN, SegNet, PSPNet, UNet and DeepLabV3+, respectively. The PA of DeepMDSCBA was 95.3%, which was 5.7%, 3.2%, 3.8%, 4.4% and 3.1% higher than that of FCN, SegNet, PSPNet, UNet and DeepLabV3+, respectively. The results of the experiments showed that the adoption of CBAM in DeepMDSCBA improved the feature extraction ability and the segmentation accuracy of various apples in the image test set, further proving the performance of the method. 

### 4.2. Efficiency of Segmentation

It can be seen in Table 7 that the training time of DeepMDSCBA was 3.52 h, which was 33%, 21%, 18% and 21% faster than that of FCN, PSPNet, UNet and DeepLabV3+, respectively. The single image prediction time of DeepMDSCBA was 32 ms, which was 42%, 15%, 26% and 42% faster than FCN, PSPNet, UNet and DeepLabV3+, respectively. DeepMDSCBA occupied 7.1 GB of memory, which was 20%, 10%, 12%, 18% and 8% less than FCN, SegNet, PSPNet, UNet and DeepLabV3+, respectively. In terms of the number of parameters, the number of parameters of DeepMDSCBA was 22.6 MB, which was 89%, 23%, 49%, 76% and 84% lower than that of FCN, SegNet, PSPNet, UNet and DeepLabV3+, respectively. However, compared with the SegNet method, the training time of DeepMDSCBA was almost 6% longer and the single image prediction time increased by 7%. This is because the SegNet method has a simpler method structure and fewer parameters than the proposed method [27], which results in a reduction in the method’s training time and single image prediction time. However, it can be seen in Figure 8 and Table 6 that the detection accuracy of SegNet was not as good as that of DeepMDSCBA. In general, because DeepMDSCBA (the method proposed in this paper) uses the lightweight network of MobileNet as its feature extraction network and changes the ordinary convolution in the ASPP module to DSC, it showed an improved calculation speed compared with the other tested methods.

### 4.3. Robustness Verification

In order to test the robustness of DeepMDSCBA, the segmentation performance of our DeepMDSCBA model trained using the training set on the four apple test sets was analyzed in comparison with the other five methods.

#### 4.3.1. Segmentation Performance of Apple Images with Different Rot Degrees

Segmentation experiments were performed on the test set of apple images with complex backgrounds, using MIoU and PA as indicators. The comparison results are shown in Table 8.

It can be seen in Table 8 that the MIoU values of DeepMDSCBA on the test set of apple images with rot degrees of (0, 20%], (20%, 40%], (40%, 60%], (60%, 80%], (80%, 100%] were 86.7%, 84.8%, 83.9%, 84.4%, and 85.1%, respectively, and the PA values were 94.4%, 92.5%, 92.2%, 92.3%, and 93.4%, respectively, which were higher than those of FCN, SegNet, PSPNet, UNet, and DeepLabV3+. Furthermore, the segmentation performance of DeepMDSCBA was better than that of the other tested models.

In addition, it can be seen in Table 8 that for all tested methods, the segmentation performance first decreased and then increased as the proportion of the rotten area increased. The PA and MIoU of each method gradually decreased until the proportion of the spoiled area was in the interval of (60%, 80%]. When the proportion of the spoiled area was in the interval of (80%, 100%], the segmentation effect was similar to that of the spoiled area in the interval of (0, 20%].

The analysis showed that with the gradual increase in the rotten area of the entire apple peel, the normal area of the apple became irregular due to the rotten area and the boundary becoming more difficult to distinguish, resulting in a gradual decrease in segmentation accuracy. When the blackened area gradually spread out to the entire apple, the overall contour of the apple and the background color could be clearly distinguished, so the segmentation accuracy again increased.

#### 4.3.2. Segmentation Performance of Apple Images with Different Rot Positions

Segmentation experiments were carried out on the test set of apple images with different rot positions using MIoU and PA as indicators. The results of the performance comparison are shown in Table 9.

It can be seen in Table 9 that the DeepMDSCBA MIoU of the test set of apple images with the view without the stalk or calyx, the calyx view, and the stalk view was 86.7%, 84.6%, and 84.7%, respectively, and the DeepMDSCBA PA was 94.4%, 92.6%, and 92.8%, respectively. These values were higher than those of FCN, SegNet, PSPNet, UNet, and DeepLabV3+, thus proving the segmentation performance of DeepMDSCBA was better than that of the other methods.

In addition, it can be seen in Table 9 that the DeepMDSCBA PA and MIoU values of the test set of apple images with the view without the stalk or calyx were higher than those of the test set of apple images with the calyx view and the stalk view. This is because the existence of the calyx and the stalk had a certain negative impact on the segmentation effect.

#### 4.3.3. Segmentation Performance of Apple Images of Different Varieties

Segmentation experiments were performed on the test set of apple images of different varieties, using MIoU and PA as indicators. The comparison results are shown in Table 10.

It can be seen in Table 10 that the MIoU of DeepMDSCBA on the test set of apple images of the Golden Delicious, Fuji, Honey Crisp and Red Delicious varieties were 87.4%, 87.6%, 87.2% and 86.8%, respectively, and the corresponding PA values were 95.4%, 95.6%, 94.9% and 94.7%, respectively; these values higher than those of FCN, SegNet, PSPNet, UNet and DeepLabV3+.

In addition, it can be seen in Table 10 that there were no significant differences among the PA and MIoU of different apple varieties using the same segmentation method, indicating that apple varieties had little effect on segmentation performance.

#### 4.3.4. Segmentation Performance of Apple Images with Complex Backgrounds

Segmentation experiments were carried out on the test set of apple images with complex backgrounds, using MIoU and PA as indicators. The comparison results are shown in Table 11.

On the apple image test set with complex backgrounds, the MIoU and PA of DeepMDSCBA were 86.8% and 94.4%, respectively, which were the highest of the tested method. The segmentation effect of DeepMDSCBA was better than FCN, SegNet, PSPNet, UNet and DeepLabV3+, and the segmentation accuracy of DeepMDSCBA was also improved.

#### 4.3.5. Segmentation Performance for Unseen Cases

To further verify the robustness of DeepMDSCBA, the following three experiments were carried out. The test set for each following experiment consisted of a certain case, which is listed in Table 5, and the training set for the experiment consisted of the other cases. The segmentation performance of DeepMDSCBA was analyzed in comparison with the other five methods.

As can be seen in Table 5, apple images with a rot degree of (40%, 60%] were used as the test set and apple images with rot degrees of (0, 20%], (20%, 40%], (60%, 80%], and (80%, 100%] were used in the training set for this experiment. The results of the performance comparison are shown in Table 12.

It can be seen in Table 12 that the MIoU and PA of DeepMDSCBA were 84.1% and 91.7%, respectively, which were also the highest of the tested methods. This means that for the apple images with an unseen rot degree, the segmentation effect of DeepMDSCBA was better than that of FCN, SegNet, PSPNet, UNet and DeepLabV3+.

For the positions of rot, apple images with the calyx view were used as the test set, and apple images with the other two views (fruit stalk view and view without the stalk or calyx) were used in the training set for this experiment. The results of the performance comparison are shown in Table 13.

The experimental results showed that the MIoU and PA of DeepMDSCBA were 84.7% and 92.3%, respectively, which were also the highest of the tested methods. The segmentation effect of DeepMDSCBA was better than that of FCN, SegNet, PSPNet, UNet and DeepLabV3+ for apple images with an unseen rot position.

For apple varieties, images of the Honey Crisp apple variety were used as the test set, and images of the Golden Delicious, Fuji and Red Delicious apple varieties were used as the training set for this experiment. The results of the performance comparison are shown in Table 14.

According to the experimental results, the MIoU and PA of DeepMDSCBA were 85.9% and 93.6%, respectively, which were still the highest of the tested methods. The segmentation effect of DeepMDSCBA was better than that of FCN, SegNet, PSPNet, UNet and DeepLabV3+ for apple images of an unseen variety.

A comparison of the experimental results of each method for each test set showed that the PA and MIoU of DeepMDSCBA were higher than those of the other tested methods, which proved that the segmentation accuracy and effect of the proposed method on all kinds of test sets were improved. At the same time, it was verified that the method had a strong generalization ability and robustness.

### 4.4. Results of Ablation Experiments

Ablation experiments were carried out based on the ablation experiment scheme described in Section 3.5.2, and MIoU and PA were used as indicators. The results of the experiments are shown in Table 15.

According to the ablation experiment results in Table 15, DeepMDSCBA had improved MIoU and PA values compared with those of DeepLabV3+, DeepM, DeepMDS, DeepMCBA, DeepMDSCBA1, and DeepMDSCBA2, indicating that replacing the backbone network with MobileNetV2 improved the segmentation accuracy of the method to a certain extent. At the same time, the MIoU and PA of DeepMCBA, DeepMDSCBA1, DeepMDSCBA2 and DeepMDSCBA were better than those of DeepM, indicating that adding CBAM to the feature extraction module or ASPP module could improve the segmentation accuracy of the method.

Furthermore, DeepMDSCBA, the method proposed in this paper, showed greater improvements than DeepMDSCBA1 and DeepMDSCBA2, indicating that adding CBAM to the feature extraction and ASPP modules at the same time could improve the segmentation accuracy of the method. DeepMDSCBA had the highest MIoU and PA values, which proved the effectiveness of this method.

Furthermore, the training segmentation efficiencies of each ablation experimental method were compared, and the results are shown in Table 16.

It can be seen in Table 16 that the training time and single image prediction time of the DeepM, DeepMDS, DeepMCBA, DeepMDSCBA1, DeepMDSCBA2 and DeepMDSCBA methods were all reduced compared with those of DeepLabV3+, indicating that the adoption of a more lightweight network (MobileNetV2) could shorten training time and improve prediction speed.

The training time and single image prediction time of the DeepMDS, DeepMDSCBA1, DeepMDSCBA2, and DeepMDSCBA methods were shorter than those of DeepLabV3+ and DeepM, indicating that changing the ordinary convolution in the ASPP module to DSC could further shorten training time and improve prediction speed.

The method training time and single image prediction time of DeepMDSCBA were the lowest of the studied methods. According to the ablation experiments, the DeepMDSCBA method proposed in this paper reduced the computational complexity of the network, shortened the training running time, and improved the segmentation accuracy.

## 5. Conclusions

A network segmentation method based on deep learning, DeepMDSCBA, was proposed in this paper. The method combines DeepLabV3+ with the optimized lightweight MobileNetV2 network and uses DSC to replace the ordinary convolution in the ASPP module, which effectively reduces the number of method parameters and improves the speed of calculation. CBAM was added to the feature extraction module and the ASPP module to better restore the edge information of objects, improve the feature extraction ability of the method, and result in fewer omissions and misclassifications. The method proposed in this paper, DeepMDSCBA, was shown to more effectively extract apple areas in images than other test methods. The PA of the whole dataset of apple images reached 95.3% and the MIoU reached 87.1%, demonstrating a more efficient and accurate segmentation of apple images compared with other tested methods, even for images of rotten apples and apples with complex backgrounds.

By comparing it with five other semantic segmentation methods on test sets of apple rot degrees, rot positions, apple varieties, and complex backgrounds, the robustness of DeepMDSCBA was fully verified. It was also proven that the performance of DeepMDSCBA was better than the other tested methods under the influence of factors such as the degree of rot, position of rot, apple variety, and complex backgrounds.

Although DeepMDSCBA’s segmentation of images of rotten apples and apples with complex backgrounds was faster and more accurate than other tested methods, its segmentation of hidden areas was not accurate for apples that were partially hidden by leaves or branches in more complex situations. It is therefore necessary to construct a relevant dataset and conduct further experimental research in the future.

## Figures and Tables

**Table 1:** MobileNetV2 network structure.

Input	Operator	t	c	n	s
256 × 256 × 3	conv2d	-	32	1	2
128 × 128 × 32	bottleneck	1	16	1	1
128 × 128 × 16	bottleneck	6	24	2	2
64 × 64 × 24	bottleneck	6	32	3	2
32 × 32 × 32	bottleneck	6	64	4	2
32 × 32 × 64	bottleneck	6	96	3	1
16 × 16 × 96	bottleneck	6	160	3	2
8 × 8 × 160	bottleneck	6	320	1	1

**Table 2:** Experimental software and hardware configuration.

Item	Detail
CPU	AMD Ryzen 7 5800H with Radeon Graphics @3.20 GHz
RAM	16 GB
Operating system	Windows 11 64-bit
CUDA	CUDA10.0
Python	Python 3.6

**Table 3:** Optimal hyperparameters.

Epoch	Batch Size	Lr	Input Shape
100	16	0.0001	512 × 512

**Table 4:** Training Set.

Training Set		Size (Before Enhancement)	Size (After Enhancement)
Rot degrees	(0, 20%]	53	159
	(20%, 40%]	57	171
	(40%, 60%]	45	135
	(60%, 80%]	47	141
	(80%, 100%]	38	114
	Subtotal	240	720
Rot positions	View without stalk or calyx	60	180
	Calyx view	60	180
	Stalk view	60	180
	Subtotal	180	540
Varieties	Golden Delicious	53	159
	Fuji	55	165
	Honey Crisp	46	138
	Red Delicious	58	174
	Subtotal	212	636
Complex backgrounds		216	648
Total		848	2544

**Table 5:** Test set.

Experiment	Test Set		Size
Performance	Total		460
Efficiency	Total		460
Robustness	Different Rot Degrees	(0, 20%]	40
		(20%, 40%]	40
		(40%, 60%]	40
		(60%, 80%]	40
		(80%, 100%]	40
		Subtotal	200
	Rot Positions	View without stalk or calyx	30
		Calyx view	30
		Stalk view	30
		Subtotal	90
	Varieties	Golden Delicious	30
		Fuji	30
		Honey Crisp	30
		Red Delicious	30
		Subtotal	120
	Complex backgrounds		50
	Unseen Cases	(40%, 60%]	40
		Calyx view	30
		Honey Crisp	30
Ablation	Total		460

**Table 6:** Performance of different apple image segmentation methods.

Method	MIoU (%)	PA (%)
FCN	82.5	90.1
SegNet	84.4	92.3
PSPNet	84.1	91.8
UNet	83.6	91.2
DeepLabV3+	84.2	92.4
DeepMDSCBA	87.1	95.3

**Table 7:** Efficiency of different apple image segmentation methods.

Method	Training Time/h	Single Image Prediction Time/ms	Memory Occupancy/GB	Parameter Quantity/MB
FCN	5.32	56	8.9	209.4
SegNet	3.34	30	8.0	20.44
PSPNet	4.43	38	8.1	44.1
UNet	4.27	43	8.7	95.3
DeepLabV3+	4.51	55	7.7	143.8
DeepMDSCBA	3.52	32	7.1	22.6

**Table 8:** Segmentation performance of apple images with different rot degrees.

Method	Indicator	(0, 20%]	(20%, 40%]	(40%, 60%]	(60%, 80%]	(80%, 100%]
FCN	MIoU (%)	82.1	80.1	79.4	80.4	81.4
	PA (%)	88.9	87.5	86.4	87.9	88.4
SegNet	MIoU (%)	83.7	82.9	82.0	82.3	83.2
	PA (%)	90.6	89.7	88.9	89.4	90.4
PSPNet	MIoU (%)	83.1	81.7	81.1	81.5	82.8
	PA (%)	89.9	88.6	88.1	88.7	89.7
UNet	MIoU (%)	82.6	81.3	80.5	81.7	82.4
	PA (%)	89.5	88.2	87.5	88.6	89.3
DeepLabV3+	MIoU (%)	83.8	82.6	81.9	82.4	83.1
	PA (%)	90.8	89.6	89.1	89.4	89.9
DeepMDSCBA	MIoU (%)	86.7	84.8	83.9	84.4	85.1
	PA (%)	94.4	92.5	92.2	92.3	93.4

**Table 9:** Segmentation performance of apple images with different rot positions.

Method	Indicator	View without Stalk or Calyx	Calyx View	Stalk View
FCN	MIoU (%)	79.9	78.4	78.6
	PA (%)	87.8	86.4	86.7
SegNet	MIoU (%)	84.6	82.7	82.5
	PA (%)	92.4	90.7	90.4
PSPNet	MIoU (%)	83.9	81.6	81.1
	PA (%)	91.7	89.4	88.4
UNet	MIoU (%)	82.1	79.6	79.2
	PA (%)	89.9	87.5	86.9
DeepLabV3+	MIoU (%)	84.8	82.5	82.2
	PA (%)	92.7	90.4	89.9
DeepMDSCBA	MIoU (%)	86.7	84.6	84.7
	PA (%)	94.4	92.6	92.8

**Table 10:** Segmentation performance of apple images of different varieties.

Method	Indicator	Golden Delicious	Fuji	Honey Crisp	Red Delicious
FCN	MIoU (%)	82.1	81.9	81.8	81.7
	PA (%)	89.8	89.6	89.5	89.4
SegNet	MIoU (%)	84.7	84.5	84.7	84.8
	PA (%)	92.4	92.2	92.6	92.8
PSPNet	MIoU (%)	83.9	83.8	83.6	83.6
	PA (%)	91.8	91.6	91.4	91.4
UNet	MIoU (%)	83.4	82.9	83.2	83.6
	PA (%)	90.9	90.5	90.7	91.3
DeepLabV3+	MIoU (%)	85.1	84.9	84.7	85.1
	PA (%)	92.8	92.6	92.4	92.9
DeepMDSCBA	MIoU (%)	87.4	87.6	87.2	86.8
	PA (%)	95.4	95.6	94.9	94.7

**Table 11:** Segmentation performance of apple images with complex backgrounds.

Method	MIoU (%)	PA (%)
FCN	80.3	87.8
SegNet	83.7	91.2
PSPNet	83.2	90.7
UNet	82.5	89.1
DeepLabV3+	84.1	90.6
DeepMDSCBA	86.8	94.4

**Table 12:** Segmentation performance for apple images with an unseen rot degree.

Method	MIoU (%)	PA (%)
FCN	78.7	86.2
SegNet	81.7	89.2
PSPNet	80.4	87.9
UNet	79.9	87.4
DeepLabV3+	82.5	89.0
DeepMDSCBA	84.1	91.7

**Table 13:** Segmentation performance for apple images with an unseen rot position.

Method	MIoU (%)	PA (%)
FCN	78.3	85.8
SegNet	82.6	90.2
PSPNet	81.5	89.1
UNet	79.7	87.3
DeepLabV3+	82.5	90.1
DeepMDSCBA	84.7	92.3

**Table 14:** Segmentation performance for apple images with an unseen variety.

Method	MIoU (%)	PA (%)
FCN	80.6	88.2
SegNet	83.5	91.1
PSPNet	82.4	90.0
UNet	82.0	89.4
DeepLabV3+	83.5	91.1
DeepMDSCBA	85.9	93.6

**Table 15:** Segmentation performance of ablation experiments.

Method	MIoU (%)	PA (%)
DeepLabV3+	83.7	92.6
DeepM	84.1	92.9
DeepMDS	84.3	93.1
DeepMCBA	85.8	94.3
DeepMDSCBA1	84.6	93.5
DeepMDSCBA2	84.8	93.8
DeepMDSCBA	87.1	95.3

**Table 16:** Segmentation efficiency of training in ablation experiments.

Method	Training Time/h	Single Image Prediction Time/ms
DeepLabV3+	4.51	55
DeepM	4.23	43
DeepMDS	3.41	34
DeepMCBA	4.23	44
DeepMDSCBA1	3.37	35
DeepMDSCBA2	3.34	38
DeepMDSCBA	3.52	32

## Data Availability

The data presented in this study are available on request from the corresponding author.
